# Both Heterozygous and Homozygous Loss‐of‐Function *JPH3* Variants Are Associated with a Paroxysmal Movement Disorder

**DOI:** 10.1002/mds.29250

**Published:** 2022-10-23

**Authors:** Dora Steel, Aikaterini Vezyroglou, Katy Barwick, Martin Smith, Julie Vogt, Frances M. Gibbon, J. Helen Cross, Manju A. Kurian

**Affiliations:** ^1^ Developmental Neurosciences, Zayed Centre for Research into Rare Disease in Children UCL Great Ormond Street Institute of Child Health London United Kingdom; ^2^ Department of Neurology Great Ormond Street Hospital London United Kingdom; ^3^ Department of Paediatric Neurology Oxford University Hospitals NHS Foundation Trust London United Kingdom; ^4^ West Midlands Regional Genetics Service Birmingham Women and Children's Hospital NHS Foundation Trust Birmingham United Kingdom; ^5^ Noah's Ark Children's Hospital for Wales Cardiff and Vale University Health Board Cardiff Wales United Kingdom

**Keywords:** alternating hemiplegia of childhood, dyskinesia, dystonia, *JPH3*, paroxysmal movement disorder

Recently, a single patient with a homozygous truncating variant in *JPH3* and a neurodevelopmental disorder involving paroxysmal dystonia was described.^1^ We report both a second individual with recessive disease and a family with a milder phenotype and autosomal dominant inheritance (Fig. 1A).

**FIG 1:**
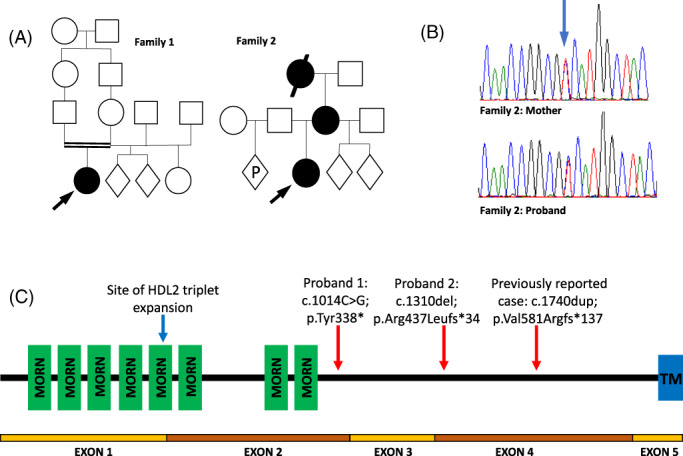
(**A**) Pedigrees for families 1 and 2. Circle: female; square: male; diamond: sex unknown; P: current pregnancy; black circle or square: affected individual; double horizontal line: consanguineous union. (**B**) Electrophoretogram of Sanger sequencing showing heterozygous variant present in patient 2 and her mother, indicated by blue arrow. (**C**) Schematic diagram of the *JPH3* gene. MORN, membrane occupation and recognition nexus repeat domain; TM, transmembrane domain. Site of previously published variants and variants reported here is indicated by arrows.

Patient one, now 15 years old, was born to first‐cousin parents with no relevant family or perinatal history. Her mother was asymptomatic, but the medical history of her father was unavailable. Early infant milestones were normal, but she did not walk until 3 years old. At school, she was identified as having moderate intellectual disability and behavioral difficulties. From 6 months old, her mother noticed episodes of abnormal body stiffening. Over time, these evolved into two clear episode types. “Minor” episodes consisted of brief right or occasionally left hemidystonia, which occurred several times each week. “Major” episodes happened two or three times per month and lasted up to 30 minutes. After a similar onset, they progressed to involuntary flailing movements of all limbs, impaired responsiveness, dysarthria, aphasia, and drooling, sometimes associated with upward eye deviation. Although “major” episodes tended to be triggered by fatigue, there was no obvious trigger for “minor” episodes. A clinical diagnosis of alternating hemiplegia of childhood (AHC) was made. Neurological examination between episodes was unremarkable. There was no disease progression over time. There was no clinical response to levodopa, oxcarbazepine, flunarizine, trihexyphenidyl, topiramate, or gabapentin, although prolonged episodes could be terminated with buccal midazolam. Extensive investigation including brain magnetic resonance imaging, electroencephalogram (EEG) (with capture of a typical episode), and cerebrospinal fluid neurotransmitters was normal. Whole genome sequencing (WGS) identified a novel homozygous frameshift in *JPH3* (NM020655.3:c.1310delG; p.Arg437Leufs*34). Parental DNA was not available for testing. The only other variant of interest was a homozygous *NDUFAF5* variant (NM_024120.5:c.524A>G; p.His175Arg), which was considered unlikely to be relevant because the phenotypic fit for mitochondrial complex I deficiency was poor and in silico tools predicted that it was probably benign.

Patient two, now 10 years old, experienced episodes of limb, facial, and orolingual dyskinesia at least weekly from the age of 6 months (Video S1). These usually started in the right upper limb before becoming generalized and lasted for several hours. No abnormal eye movements were evident during the episodes, and there was no obvious trigger. Between episodes, very mild resting dyskinesia was evident in the upper limbs. She had moderate learning difficulties, but no behavioral issues. Again, extensive investigation was unremarkable, including EEG with capture of episodes. Several different medications—carbamazepine, valproate, topiramate, acetazolamide, and trihexyphenidyl—proved ineffective. Her maternal grandmother had experienced lifelong episodes of orofacial dyskinesia and limb posturing and had never learned to read. Her mother reported episodes of orofacial dystonia triggered by alcohol and had a slight resting tremor. She had completed compulsory education. The only relevant variant identified on WGS was a novel heterozygous nonsense variant in *JPH3* (NM020655.3:c.1014C>G; p.Tyr338*) shared by mother and daughter: grandmaternal DNA was unavailable (Fig. 1B).

*JPH3* encodes junctophilin‐3, a component of the junctional complex linking the plasma membrane with the endoplasmic reticulum in excitable cells.^2^ Expression is highest in the brain. Pathological heterozygous triplet‐repeat expansion variants (believed to cause toxic intracellular accumulation of abnormal protein)^3^, ^4^ occur in Huntington‐like disease type 2, characterized by adult‐onset chorea, dementia and atrophy of the cortex, and basal ganglia.^5^ However, we believe that the disorder from either biallelic or heterozygous truncating variants is a distinct condition, mediated by loss of protein function rather than toxic accumulation, and so far, appears non‐progressive in reported cases (Fig. 1C). The gene is predicted to be highly intolerant of loss‐of‐function,^6^ and in mice, both haploinsufficiency and knockout of *JPH3* result in an abnormal motor phenotype.^7^ Although the association of the variably penetrant, heterozygous phenotype will require further confirmation in additional patients, *JPH3* variants should be considered in patients with undiagnosed complex paroxysmal movement disorders.

## Financial Disclosures

None of the authors has any financial disclosures relevant to the research. Financial relationships are listed below.

D.S. is employed by UCL with a salary paid by a grant from the National Institute for Health Research. A.V. is employed by UCL. K.B. is employed by UCL. M.S. is employed by the Oxford Radcliffe Hospitals National Health Service (NHS) Foundation Trust. J.V. is employed by the Birmingham Women and Children's Hospitals NHS Foundation Trust. F.G. is employed by the Cardiff and Vale University Health Board. H.C. is employed by UCL with grants from various organizations. M.A.K. is employed by UCL. M.A.K.’s research group receives grants from the National Institute of Health Research; Rosetrees Fund; Great Ormond Street Hospital Charity; LifeArc; and the Sir Jules Thorn Trust.

## Author Roles

(1) Research project: A. Conception, B. Organization, C. Execution; (2) Statistical Analysis: A. Design, B. Execution, C. Review and Critique; (3) Manuscript: A. Writing of the First Draft, B. Review and Critique.

D.S.: 1A, 1C, 3A

A.V.: 1C, 3B

K.B.: 1C, 3B

M.S.: 3B

H.C.: 3B

M.A.K.: 1A, 1B, 3B

## Funding Information

Professor Kurian's laboratory receives funding from the National Institute for Health Research and the Sir Jules Thorn Charitable Trust.

## Supporting information


**Video S1** Dyskinetic episodes experienced by patient 2. Segment 1: patient is approximately 3 years old, and several hours into the episode: note her marked orofacial dyskinesia and the need for truncal support. Segment 2: patient is approximately 5 years old. Note marked generalized hyperkinetic/flailing movements. Segment 3: patient is 9 years old. Despite generalized dyskinesia, she is comfortable and eating an ice lolly.

## Data Availability

We are unable to make patients' full genomic data available under the terms of our ethical approval but are happy to share details of methods and laboratory or clinical results on request.
